# Orthotopic Heart Transplantation following univentricular palliation: new challenges for the congenital cardiac surgeon

**DOI:** 10.1186/1749-8090-10-S1-A92

**Published:** 2015-12-16

**Authors:** María-Teresa González-López, Juan-Miguel Gil-Jaurena, Ramón Pérez-Caballero-Martínez, Ana-María Pita-Fernández, Manuela Camino-López, Nuria Gil-Villanueva, José-Luis Zunzunegui-Martínez, Constancio Medrano-López

**Affiliations:** 1 Paediatric Cardiac Surgery Department, Gregorio Marañón Hospital, Madrid, Spain; 2 Paediatric Cardiology Department, Gregorio Marañón Hospital, Madrid, Spain

## Background/Introduction

Orthotopic heart transplantation after univentricular palliation presents a difficult challenge due to the complex anatomy and prior surgeries.

## Aims/Objectives

We present our surgical techniques/results in the current era.

## Method

2013-2014: 23 congenital cardiac patients underwent heart transplantation. 13 of them: previous univentricular palliation: hypoplastic left heart syndrome (n = 8), d-transposition of great arteries+criss-cross-heart (n = 1), unbalanced atrioventricular septal defect (n = 1), pulmonary atresia+intact ventricular septum (n = 1) and grown-up patients (GUCH) (n = 2: 1 double-inlet-left-ventricle and 1 tricuspid atresia).

## Results

Paediatric group (n = 11): age 7.1+/-4.8 years (range 1.5 months-13 years); weight 21.3+/-9.5 kilograms (range 3.5-36). GUCH-group (n = 2): age 23.5+/-0.5 years; weight 50+/-10.2 kilograms. 46.1% had undergone Fontan completion, 15.4% Fontan take-down, 30.7% bidirectional cavopulmonary shunt and 7.7% Blalock-Taussig shunt. Berlin-Heart-EXCOR-Paediatric-Device as bridge to transplantation was used in 1 patient. Bicaval technique was performed along with: hemiarch repair (15.3%,n = 2), pulmonary artery (PA) branches plasty (38.4%,n = 5), hilum-to-hilum PA reconstruction (53.8%,n = 7), superior venae cavae reconstruction (15.4%, n = 2) and stent removal from PA (61.5%,n = 8), inferior venae cavae (7.7%,n = 1) and lateral-tunnel-Fontan (7.7%,n = 1). Average cardiopulmonary-bypass time 257.6+/-79.3 minutes (range 120-431); total-ischemia-time 220.7+/-48.6 (range 140-287). One patient required ECMO; 4 underwent delayed sternal closure; 2 underwent diaphragm plication; 1 subacute-humoral-rejection treated with plasmapheresis. In-hospital stay 44+/-16 days (range 18-185). At follow-up (14.4+/-7.2 months), freedom from percutaneous procedures 83.3% (n = 10). 30-day mortality/follow-up mortality: zero. All of them remain with an optimal functional class.

## Discussion/Conclusion

Heart transplantation following univentricular palliation is technically demanding but short-term results are excellent. An extensive surgical reconstruction (donor/heterologous tissues) is mandatory to improve outcomes. Further follow-up is necessary to evaluate the long-term results in this scenario.

